# Paramyosin of canine *Onchocerca lupi*: usefulness for the diagnosis of a neglected zoonotic disease

**DOI:** 10.1186/s13071-016-1783-z

**Published:** 2016-09-07

**Authors:** Bronwyn Campbell, Helder Cortes, Giada Annoscia, Alessio Giannelli, Antonio Parisi, Maria Stefania Latrofa, Filipe Dantas-Torres, Luís Cardoso, Domenico Otranto

**Affiliations:** 1 Dipartimento di Medicina Veterinaria, Universitá degli Studi di Bari, Bari, Italy; 2 Victor Caeiro Laboratory of Parasitology, Instituto de Ciências Agrárias e Ambientais Mediterrânicas, Universidade de Évora, Évora, Portugal; 3 IZS Istituto Zooprofilattico Sperimentale Puglia e Basilicata, Putignano, Italy; 4 Departamento de Imunologia, Centro de Pesquisas Aggeu Magalhães (Fiocruz-PE), Recife, Brazil; 5 Department of Veterinary Sciences, School of Agrarian and Veterinary Sciences, University of Trás-os-Montes e Alto Douro (UTAD), Vila Real, Portugal

**Keywords:** *Onchocerca lupi*, Zoonotic, Paramyosin, Bioinformatics, Diagnostic tests, Vaccine

## Abstract

**Background:**

Of increasing importance to the medical and veterinary communities is the zoonotic filarioid nematode *Onchocerca lupi.* Onchocercosis, thus far found in wolves, dogs, cats and humans, is diagnosed *via* skin snips to detect microfilariae and surgical removal of adults from the eye of the host. These methods are time-consuming, laborious and invasive, highlighting the need for new tools for the diagnosis of *O. lupi* in susceptible hosts. Symptoms related to the presence of the adults in the eye can range from none apparent to severe, including blindness. No reliable chemotherapeutic protocols are available, as yet, to eliminate the infection. Paramyosin, an invertebrate-specific protein, has been well-studied as an allergen, diagnostic marker and vaccine candidate. The aim of this study, therefore, was to isolate and characterise paramyosin from *O. lupi* to assess its suitability for the development of a serological diagnostic assay.

**Methods:**

The adult and microfilarial stages of *O. lupi* were isolated from the eyes and skin of a 3-year-old male dog. Total RNA was extracted and reverse transcribed into single stranded cDNA. Reverse-transcription PCR was used to isolate a full-length paramyosin cDNA from adult worms and to investigate the temporal expression patterns of this gene. All amplicons were sequenced using dideoxy chain termination sequencing. Bioinformatics was used to predict the amino acid sequence of the gene, to compare the DNA and protein sequences with those available in public databases and to investigate the phylogenetic relationship of all molecules. Antibody binding sites were predicted using bioinformatics and mapped along with published antigenic epitopes against the *O. lupi* paramyosin protein. The native protein, and three smaller recombinantly expressed peptides, were subjected to western blot using serum from dogs both positive and negative for *O. lupi*.

**Results:**

Paramyosin of *O. lupi* was herein molecularly characterized, encoded by a transcript of 2,643 bp and producing a protein of 881 amino acids (101.24 kDa). The paramyosin transcript was detected, by reverse transcription PCR, in adults and microfilariae, but not in eggs. Phylogenetic analysis indicates that this molecule clusters with paramyosins from other filarioids to the exclusion of those from other taxa. A total of 621 unique antibody binding epitopes were predicted for this protein and another 28 were conserved in other organisms. This information was used to design three peptides, for recombinant expression, to identify the antibody binding epitope(s) and reduce potential cross-reactivity with serum from dogs infected with other filarioid nematodes. Native paramyosin, purified from microfilariae and adults, was detected by antibodies present in serum from dogs with known *O. lupi* infections.

**Conclusions:**

Data provided herein may assist in the development of a serological diagnostic test, based on antibodies to *O. lupi* paramyosin, for the diagnosis of this infection, in order to gain more information on the real distribution of this little known filarioid of zoonotic concern.

**Electronic supplementary material:**

The online version of this article (doi:10.1186/s13071-016-1783-z) contains supplementary material, which is available to authorized users.

## Background

Ocular parasitism in humans and animals may be caused by a range of zoonotic helminths, including strongylids, ascarids, thelaziids and filarioids [1]. The parasitic nematode *Onchocerca lupi* (Spirurida: Onchocercidae) localises to the connective tissue of the sclera of dogs, wolves and cats [2]. In dogs, subconjunctival granulomas are the most commonly reported manifestation of infection by this filarioid [3]; however, clinical signs may vary from none apparent [4] to severe (i.e. blindness) [2]. Affected dogs have periodically been reported from Hungary, Greece, Germany, Portugal and Spain [3, 5–10]. More recently, reports have also emerged of infections of dogs and cats in the United States of America [11, 12].

The suspected zoonotic potential of *O. lupi* [2, 8, 9, 11, 13] has only recently been confirmed [14–16]. To date, human ocular infection has been documented in Turkey [14, 17], Tunisia [15], Iran [18] and the United States of America [19]. Three additional cases, where localisation of *O. lupi* is to the cervical spine, have been reported in children in the USA [19–21], prompting an increased interest in this nematode by the medical and veterinary communities [22].

There is a paucity of information on the biology and epidemiology of *O. lupi* (e.g. vector insects and geographical distribution). The only report available, to date, on the prevalence of *O. lupi* came from apparently healthy dogs sampled in Greece (8.7 %) and Portugal (8.3 %) [4]. In addition, the DNA sequence data available are limited to single ribosomal and mitochondrial genes, or parts thereof, which have been used for molecular and phylogenetic analyses [23–25]. Importantly, the frequency, distribution and full zoonotic potential of this parasite has not yet been investigated, probably due to the difficulty in achieving a diagnosis of the infection, which is based on the detection of microfilariae in skin sediments or surgical removal and identification of the adult worm from the eye [26]. Some preliminary scientific evidence indicates that an ELISA, prepared with somatic paramyosin antigens from *Onchocerca gibsoni* (Og4C3), which infects cattle [27–29], cross-reacts with serum from dogs infected with *O. lupi* [30].

Paramyosin is a large structural protein (98–101 kDa) component of invertebrate muscle and found in endo- and ectoparasites of medical importance, but not in vertebrates [31]. By virtue of its major antigenic properties, this protein has been used for the development of diagnostic assays for filarioids of medical concern such as *Wuchereria bancrofti* and *Brugia malayi* [27, 32, 33]. Many studies of the paramyosin protein, as a potential vaccine candidate, have focussed on platyhelminths [34–39], with fewer studies on nematodes [40, 41] and ticks [42]. An immunomodulatory effect of this protein has also been identified, inhibiting components of the complement cascade [43, 44], binding of immunoglobulins and collagen [45, 46]. This suggests paramyosin is an important part of the mechanism by which a parasite invades and maintains itself in the host [31], making this protein of potential usefulness for serological diagnosis of, and protective immunity against, *O. lupi*.

Therefore, the research herein describes the isolation and characterisation of a paramyosin transcript from *O. lupi*, bioinformatic analysis of the predicted protein and preliminary assessment of the potential for the full protein and recombinant pieces for the development of a specific serological diagnostic assay.

## Methods

### Biological material

Three adult female and 2 adult male nematodes were isolated from the eye of an infected dog (3-year-old, male) that died accidentally, from an area (Algarve, southern Portugal) where *O. lupi* is known to occur [4]. Microfilariae were collected by sedimentation from the skin of the dorsal region of the dog and eggs by cutting the uterus of the adult females. All samples were preserved in RNAlater® (Life Technologies, California, USA) and stored at -80 °C until used. Serum samples were obtained from this dog and from *O. lupi* positive (*n* = 5) and negative dogs (*n* = 3) from previous research [30].

### Nucleic acid isolation and single-stranded cDNA synthesis

Total RNA was isolated from adult and microfilarial *O. lupi*, following homogenization using a pestle and 400–600 μm glass beads (Sigma-Aldrich, Missouri, USA) employing the TriPure® isolation reagent according to the manufacturer's instructions (Roche Molecular Biochemicals, Basel, Switzerland). RNase inhibitor (RNasin®, Promega, Wisconsin, USA) was added to total RNA before quantification and storage. Due to the tiny amounts of RNA able to be extracted from these nematodes, no DNase treatment was performed. Nucleic acids were quantified using a Bioanalyzer (Agilent, California, USA) and all extracted material was stored at -80 °C until used. First-strand cDNA synthesis was performed using the SuperScript Reverse Transcriptase II kit (Invitrogen, California, USA), 0.5 g oligo dT primer (*n* = 12–18 primer, Promega, Wisconsin, USA) and 100 ng of total RNA from adult males and females, microfilariae and eggs according to the manufacturer’s instructions. Each completed reaction was diluted to 250 ng/l with TE (10 mM Tris-HCl, 1 mM EDTA, pH 8.0) before use in reverse transcription PCR.

### PCR of the paramyosin cDNA

PCR of the paramyosin cDNA, using 250 ng of single stranded cDNA from a single adult female, was performed using Phusion High-Fidelity DNA polymerase (Life Technologies, California, USA) and primers (PARAdegF 5′-ATG TCC GGT TCA TTG TAC CGT AG-3′; PARAdegR 5′-CTA TTG CTC ATC TTC GAG AAC GT-3′), designed from available filarial nematode paramyosin sequences (*B. malayi* NCBI Accession no. U77590.1, *Onchocerca volvulus* NCBI Accession no. M95813.1 and *Acanthocheilonema viteae* nAv_1_0_scaf01040, www.nematodes.org). Reactions consisted of 1× GC reaction buffer, 2 pmol of each primer, 0.2 mM of dNTPs and 1 U of Phusion Taq DNA polymerase in a volume of 50 μl. Cycling conditions were 98 °C/10 s for initial denaturation, then 35 cycles of 98 °C for10 s, 55 °C for 20 s and 72 °C for 3 min for DNA amplification and 72 °C for 10 min for the final extension. Products were resolved on 1 % agarose stained with 0.5× GelRed (Biotium, California, USA) and visualized on a GelLogic 100 gel documentation system (Kodak, New York, USA). Amplicon sequencing employed the PCR primers and Big Dye Terminator v.3.1 chemistry in a 3130 genetic analyzer (Applied Biosystems, California, USA).

### Gene cloning and sequencing

The full-length cDNA isolated above was cloned into pGEM®-T Easy, employing T4 DNA ligase (3 U), and transformed into *Escherichia coli* (MACH-1 strain, Life Technologies, California, USA) using an established method [47]. Colonies were picked, resuspended in 20 μl of water, with 2 μl used as a PCR template for insert screening and the remaining grown overnight in Luria Bertani/ampicillin (100 g/ml) broth. Plasmid DNA from positive colonies was isolated using the PureYield™ Plasmid Miniprep System (Promega, Wisconsin, USA) and inserts were sequenced in both directions using the T7 and SP6 primers of pGEM®-T Easy [47].

### Temporal expression of *O. lupi* paramyosin in adult males and females, microfilariae and eggs

The entire paramyosin (2,643 bp; PARA_degF, PARA_degR primers) and elongation factor 1α (as a control gene, designed from the *O. volvulus* ELF1α (1,776 bp; GenBank M64333.1, OvELF1α_F 5′-ATT GAG ATT TCG GGA TTA AGT GAA T-3′, OvELF1α_R 5′-TTC AGT GTA GCA GGA GCA TAT G-3′) genes were amplified using Phusion High-Fidelity DNA polymerase enzyme (Life Technologies, California, USA) according to the method used above for cDNA amplification (53 °C annealing for ELF1α). Reactions used 125 ng of single-stranded cDNA template for adults and eggs and 500 ng for microfilariae. Two reactions were performed for microfilariae using template from the first reaction, including the negative controls, in order to amplify both genes. The experiment was repeated 3 times (i.e. 3 replicates per gene) on 3 separate days. Products were sequenced using the PCR primers as described above.

### Bioinformatic analyses and epitope mapping

The identity of the full-length *O. lupi* paramyosin cDNA was confirmed using BLASTx/n/p against nucleotides and proteins available in NCBI (blast.ncbi.nlm.nih.gov) with an E-value limit of 1e^-05^. The predicted *O. lupi* paramyosin protein was subjected additional analyses, including ProSite (prosite.expasy.org), the presence of signal peptides (http://www.cbs.dtu.dk/services/SignalP/; [48–50], transmembrane spanning domains (www.cbs.dtu.dk/services/TMHMM/) [51] and detection of conserved protein domains (ProSite; www.expasy.ch/tools/scnpsit1.html). Potential antigenic epitopes were predicted using algorithms based on published methods [52–55]. Epitopes reported in the literature for other organisms (Table 1) and bioinformatically predicted epitopes were mapped against the *O. lupi* protein using local BLAST+ (NCBI). Epitopes unique to *O. lupi* paramyosin were identified through comparisons with epitopes predicted for other filarial nematodes, namely *A. viteae* (bioinformatically predicted, see below)*, Ancylostoma caninum* (NCBI Accession no. ABC86903.1)*, B. malayi* (NCBI Accession no. Q01202.2)*, Dirofilaria immitis* (NCBI Accession no. P13392.2)*, Loa loa* (PantherDB Accession no. E1FX82), *O. volvulus* (NCBI Accession no. M95813.1) and *W. bancrofti* (NCBI Accession no. AEY79495.1). Paramyosin genes/proteins from other organisms were identified by BLASTp of non-redundant (NCBI, www.pantherdb.org/, www.metazoa.ensembl.org) and parasite-specific databases (www.broadinstitute.org/, www.nematodes.org/, www.nematode.net/, www.wormbase.org). Augustus gene prediction, using *B. malayi* as the training set, was used to predict the full-length genes and proteins from genomic data (http://bioinf.uni-greifswald.de/augustus/) [56]. These were confirmed *via* BLASTn/p against the NCBI database. All paramyosin molecules were aligned using MUSCLE [57] and full-length sequences subjected to phylogenetic analyses by Bayesian inference (MrBayes v 3.2.6) probabilities were calculated using 100,000 generations (ngen = 100,000, burnin = 20), employing 4 simultaneous tree-building chains (nchains = 4), saving every 100 th tree (samplefreq = 100). The heuristic neighbour-joining (NJ) analysis was performed employing MEGA 7.0 using the absolute difference model (2,000 replications) with the alignment gaps treated as missing data. The protein sequence from *Meloidogyne hapla* (www.wormbase.org; Accession number: Contig185) was assigned as the outgroup.

**Table 1:** Empirically determined paramyosin epitopes interacting with components of the immune system of various organisms (nematodes, platyhelminths and arthropods)

Species	Type	Epitopes detected (amino acids)	Part of the protein	Reference
*Haemaphysalis longicornis*	-	L71-E92	N-terminal	[42]
*Onchocerca volvulus*	B-cell	A253-D265	N-terminal	[62]
*Schistosoma japonicum*	B-cell	I363-A366	Middle	[63]
		L362-A366		
*Schistosoma mansoni*	T- and B-cell	M1-T25	Whole	[72]
		L481-E500		
		N355-K367		
	T-cell	T6-P22	N-terminal	[64]
		N366-D382	Middle	
		Y210-S226		
	Complement	A744-M866	C-terminal	[43]
	B-cell	G19-L27	Whole	[72]
		L66-G71		
		D93-N98		
		L155-E166		
		K232-A239		
		E275-H283		
		E305-E312		
		E326-A338		
		R372-E377		
		Q400-L404		
		L460-L467		
		R502-E511		
		R579-A590		
		L686-H695		
		E766-I774		
		Q798-E804		
		R814-M829		
		L852-G859		
	T-cell	V10-Y17	Whole	[72]
		Q58-R65		
		V123-D136		
		L178-L186		
		L196-E211		
		Q259-E275		
		L439-R446		
		T516-Y524		
		V630-V637		
		K787-A794		
		V805-K812		
*Taenia solium*	-	R10-T16	N-terminal	[65]
	T-cell	D176-L192		[66]
*Trichinella spiralis*	Antibody	E88-E107	N-terminal	[67]

### Protein expression and western blots

Native paramyosin was isolated from microfilariae and adults using a differential ammonium sulphate precipitation method [58]. Protein was measured using the Qubit® Protein Assay Kit according to the manufacturer’s instructions (Thermo Fisher Scientific, Massachusetts, USA). Subsequently, in order to define antigenic epitope(s) recognized by antibodies from dogs infected with *O. lupi*, bioinformatics was used to design 3 peptides, corresponding to both the presence of potential antigenic epitopes and natural methionine residues (to maintain the correct reading frame). These sections were designated pieces 1 (1,272 bp, 48.2 kDa), 2 (908 bp, 33.5 kDa) and 3 (513 bp, 19.65 kDa) from the N- to the C-terminal part of the protein respectively. Primers used were: Piece 1 - PARAdegF, Piece 1R 5′-CGC CTT CTC GTA CAA ATT CTT CAT-3′; Piece 2 - PIECE2F 5′-ATG AAG AAT TTG TAC GAG AAG GCG-3′, PIECE2R 5′-GAT TTC CTG AGA GCA TCA ATT TTC AT-3′; Piece 3 - PIECE3F 5′-ATG AAA ATT GAT GCT CTC AGG AAA TC-3′, PARAdegR using Phusion Taq polymerase under the conditions described above. All cDNAs were ligated into the SmaI restriction enzyme site of the pGEX3X glutathione S-transferase fusion protein expression vector (GE Healthcare Bio-Sciences, Pennsylvania, USA) using T4 DNA ligase (3 U) and transformed into *E. coli* (BL21 strain). Clones were induced using 1 mM isopropyl β-D-1-thiogalactopyranoside (IPTG) for 3 h and then analyzed *via* SDS PAGE (10 % gels) using an established protocol [47]. Expressed peptides were purified using EZview™ Red Glutathione Affinity Gel (Sigma Aldrich, Missouri, USA) according to the manufacturer’s instructions, without elution of the peptide from the resin. Peptides were then cleaved from GST using Factor Xa protease (0.5 U, Promega, Wisconsin, USA) and the cleaved peptides isolated from the supernatant. Following SDS PAGE (10 % gels) of total nematode protein (positive control), purified native paramyosin (< 0.5 ng - microfilariae, 500 ng - adult), purified recombinant peptide (500 ng), uninduced bacterial culture (5 μl) and the Precision Plus Protein™ All Blue Prestained Protein Standards (BioRad, California, USA), proteins were transferred to PVDF membrane (0.5A/45 min; Roche Applied Sciences, Penzburg, Germany). Following blocking, membranes were incubated with serum from uninfected dogs (negative control) or those infected with *O. lupi* (1:1000 dilutions). Dog antibodies were detected using rabbit anti-dog IgG horseradish peroxidase conjugate (1:3000; Sigma Adrich, Missouri, USA) and detected using the *femto-*Chromo kit (G Biosciences, Missouri, USA). Documentation was *via* scanning on an Epson Perfection 2450 scanner.

## Results

The full-length cDNA encoding paramyosin (henceforth named *Ol-para*) is 2,643 bp in length (NCBI Accession no. KJ699378). BLASTn analysis revealed the highest nucleotide identity to paramyosin from the related filarial nematode *O. volvulus* (98 %, EMBL Accession no. M95813.1), followed by *L. loa* (91 %, NCBI Accession no. JH712727.1) and *B. malayi* (90 %, NCBI Accession no. U77590.1). Isolation and alignment of the nucleotide sequences above, and from other organisms, and comparison with *Ol-para* revealed that the percentage identity with platyhelminths was, on average, 52 %. Non-filarioid nematodes showed an average nucleotide identity with *Ol-para* of 77 %, whilst the highest nucleotide identities were with the other filarioid nematodes, except *W. bancrofti* (52 %) (Table 2). Analysis of the nucleotide composition of *Ol-para* and other filarioid nematode sequences indicated that all are AT-rich (*Ol-para*, 60.23 %) (Table 2). The conceptually translated protein (*Ol*-PARA) was 881 amino acids in length, with a predicted molecular weight of 101.24 kDa (NCBI Accession no. AJO15918.1). Amino acid identities with *O. volvulus*, *L. loa* and *B. malayi* were 93 % for the first 2 and 90 %, for the latter. No signal peptides or transmembrane spanning domains were identified in *Ol*-PARA, consistent with the other nematode paramyosins analyzed. Conserved protein domain analysis revealed the presence of a proton donor site in *Ol*-PARA (and in some, but not all, of the other nematode proteins).

**Table 2:** Percentage nucleotide identity matrix between full-length paramyosin sequences (accession numbers in the matrix) of nematode and platyhelminth species. The accession numbers, length of sequence (bp) and AT-richness of the sequences are also indicated. The database from which the DNA sequences were retrieved are indicated

Species	Accession number	Length (bp)	% nucleotide identity with *Ol-para*	% AT
*Acanthocheilonema viteae* ^a^	nAv_1_0_scaf01040	2,511	92	60
*Ancylostoma caninum* ^b^	DQ358106.1	2,634	71	44
*Anisakis pegreffii* ^b^	JX986981.1	2,610	77	54
*Anisakis simplex* ^b^	AF173004.1	2,610	77	54
*A. simplex* ^b^	JX986984.1	2,610	77	54
*Ascaris suum* ^b^	AG01283	2,610	74	48
*A. suum* ^b^	JI170788.1	1,575	77	50
*Brugia malayi* ^*b*^	U77590.1^b^	2,643	91	61
*Caenorhabditis elegans* ^*b*^	F07A5.7a^c^	2,619	72	47
*C. elegans* ^*b*^	F07A5.7b.1^c^	1,662	73	48
*Clonorchis sinensis* ^b^	JQ041818.1	2,595	56	51
*C. sinensis* ^b^	EF071860.1	2,595	56	51
*Dictyocaulus viviparus* ^b^	EF426295.1	2,631	77	54
*D. viviparus* ^b^	EU044828.1	2,631	77	53
*D. viviparus* ^b^	EU044829.1	2,631	76	53
*Echinococcus granulosus* ^b^	CAA79849.1	1,000	75	49
*E. granulosus* ^b^	Z21787.1	2,592	53	47
*Loa loa* ^b^	JH712727.1	2,637	91	61
*Onchocerca volvulus* ^b^	M95813.1	2,640	98	61
*Paragonimus westermani* ^b^	AAY44740.1	2,595	56	53
*Schistosoma japonicum* ^b^	AF113971.1	2,662	58	65
*S. japonicum* ^b^	EU488865.1	2,601	59	65
*Schistosoma mansoni* ^b^	M35499.1	3,156	61	67
*Taenia saginata* ^b^	AJ439882.1	3,408	57	48
*Taenia solium* ^b^	AY034087.1	2,592	52	46
*Wuchereria bancrofti* ^b^	JN639883.1	2,619	59	60

^a^ nematodes.org

^b^ ncbi.nlm.nih.gov

**Fig. 1:**
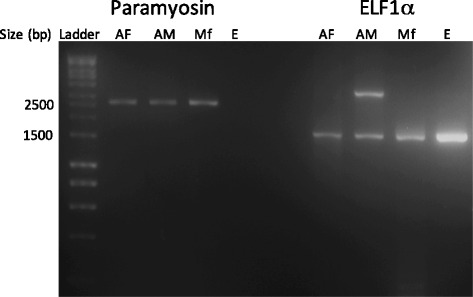
Reverse transcription PCR of *Onchocerca lupi* paramyosin (2,643 bp) and of the control gene elongation factor 1α (1,776 bp, ELF1α). The 2.5 kb and 1.5 kb bands from the 1 kb DNA ladder used as the standard are indicated on the left for reference. *Abbreviations*: AF, adult female; AM, adult male; Mf, microfilariae; E, eggs

Reverse transcription PCR (RT-PCR) amplified *Ol-para* from adult males and females, microfilariae, but not from eggs, whereas ELF1α was amplified from all stages (Fig. 1). Sequencing and BLASTn analysis of *O. lupi* ELF1α indicated a 94 % nucleotide identity to *O. volvulus* ELF1α (NCBI Accession no. M64333.1).

**Fig. 2:**
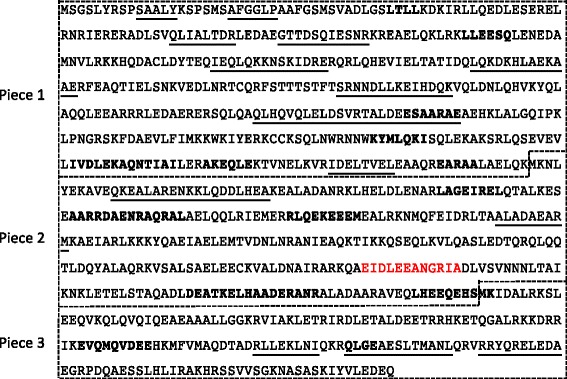
Immunologically reactive paramyosin epitopes reported in the literature for helminths and arthropods that are conserved in the *Onchocerca lupi* protein. Residues underlined indicate T-cell epitopes whilst those in *bold* indicate B-cell epitopes. The text in *red* indicates residues of the proton donor site. The paramyosin peptides designed using bioinformatics to detect the antibody binding epitopes are indicated using dashed line boxes

A total of 874 antibody binding epitopes, predicted using bioinformatic algorithms, and 14 published T-cell and 14 B-cell epitopes were mapped against *Ol*-PARA (Fig. 2 and Additional file 1: Figure S1). Of the 874 predicted antibody epitopes, when compared with those predicted for *A. viteae*, *B. malayi*, *D. immitis*, *L. loa*, *O. volvulus* and *W. bancrofti*, 621 were unique to *O. lupi*.

**Fig. 3:**
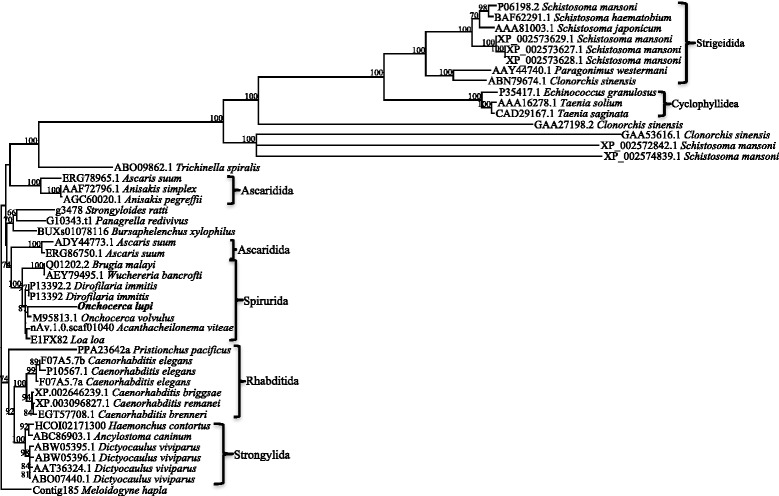
Phylogenetic relationships among nematode and platyhelminth paramyosin proteins. Forty-six full-length paramyosin proteins from a range of nematodes and platyhelminths were aligned and subjected to phylogenetic analysis. The topology of the trees from Bayesian inference and maximum parsimony analyses were in consensus, the representative tree displayed here with bootstrap values assigned to each branch (Bootstrap cut-off of 50). The protein sequence from *Meloidogyne hapla* (www.wormbase.org; Accession number: Contig185) was assigned as the outgroup

**Fig. 4:**
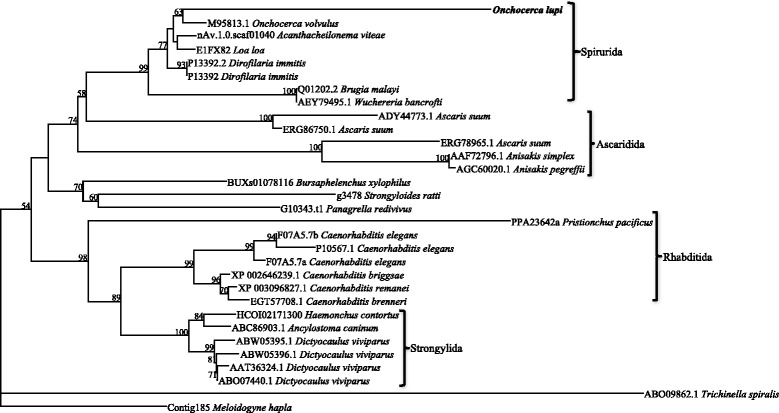
Phylogenetic analysis of nematode proteins. Thirty-two full-length paramyosin proteins from a range of nematodes were aligned and subjected to phylogenetic analysis. The topology the of trees from Bayesian inference and maximum parsimony analyses were in consensus, the representative tree displayed here with bootstrap values assigned to each branch (Bootstrap cut-off of 50). The protein sequence from *Meloidogyne hapla* (www.wormbase.org; Accession number: Contig185) was assigned as the outgroup

Forty-six full-length paramyosin molecules from a range of nematodes and platyhelminths were identified, some species having more than one form of paramyosin (Table 3). Alignment and phylogenetic analysis show distinct clusters corresponding to nematodes and platyhelminths (Fig. 3). In the nematode branch, free-living nematodes such as *Caenorhabditis* spp. grouped together, whilst the parasitic nematodes formed two separate groups. The Platyhelminthes consist of both digeneans and cestodes, each forming a separate group. Further phylogenetic analysis of the nematode paramyosin molecules separates the nematode proteins according to distinct taxonomic groups (Fig. 4).

**Table 3:** Species for which full-length paramyosin protein sequences were used in the bioinformatic and phylogenetic analyses, including length (amino acids), accession number and the database from which the sequence was retrieved. Sources of sequences are also reported

Species	Accession number	Length (aa)
*Acanthocheilonema viteae* ^a, e^	nAv_1_0_scaf01040	836
*Ancylostoma caninum* ^b^	ABC86903.1	877
*Anisakis pegreffii* ^b^	AGC60020.1	869
*Anisakis simplex* ^b^	AAF72796.1	869
*Ascaris suum* ^b^	ADY44773.1	524
*A. suum* ^b^	ERG78965.1	869
*A. suum* ^b^	ERG86750.1	919
*Brugia malayi* ^b^	U77590.1	880
*Bursaphelenchus xylophilus* ^c^	BUX s01078	882
*Caenorhabditis brenneri* ^c, e^	EGT57708.1	838
*Caenorhabditis briggsae* ^b^	XP 002646239.1	872
*Caenorhabditis elegans* ^c^	F07A5.7a	872
*C. elegans* ^c^	F07A5.7b	553
*Caenorhabditis remanei* ^b^	XP 003096827.1	872
*Clonorchis sinensis* ^b^	ABN79674.1	864
*C. sinensis* ^b^	GAA27198.2	934
*C. sinensis* ^b^	GAA53616.1	979
*Dictyocaulus viviparus* ^b^	AAT36324.1	876
*D. viviparus* ^b^	ABO07440.1	876
*D. viviparus* ^b^	ABW05395.1	876
*D. viviparus* ^b^	ABW05396.1	876
*Dirofilaria immitis* ^b^	P13392.2	874
*D. immitis* ^b^	P13392	875
*Loa loa* ^d^	E1FX82	879
*Echinococcus granulosus* ^b^	P35417.1	863
*Haemonchus contortus* ^c, e^	HCOI02171300	765
*Meloidogyne hapla* ^c, e^	Contig185	648
*Onchocerca volvulus* ^b^	M95813.1	879
*Panagrella redivivus* ^c, e^	G10343	876
*Paragonimus westermani* ^b^	AAY44740.1	864
*Pristionchus pacificus* ^b^	PPA23642	767
*P. pacificus* ^d^	H3FEM5	939
*Schistosoma haematobium* ^b^	BAF62291.1	866
*Schistosoma japonicum* ^b^	AAA81003.1	866
*Schistosoma mansoni* ^b^	P06198.2	866
*S. mansoni* ^b^	XP 002572842.1	1228
*S. mansoni* ^b^	XP 002573627.1	1124
*S. mansoni* ^b^	XP 002573628.1	1068
*S. mansoni* ^b^	XP 002573629.1	816
*S. mansoni* ^b^	XP 002574839.1	807
*Strongyloides ratti* ^c, e^	G3478	881
*Taenia saginata* ^b^	CAD29167.1	863
*Taenia solium* ^b^	AAA16278.1	863
*Trichinella spiralis* ^b^	ABO09862.1	885
*Wuchereria bancrofti* ^b^	AEY79495.1	880

^a^ nematodes.org

^b^ ncbi.nlm.nih.gov

^c^ www.wormbase.org

^d^ www.pantherdb.org

^e^Full-length proteins predicted *via* bioinformatic analysis (genome mining, Augustus gene prediction)

**Fig. 5:**
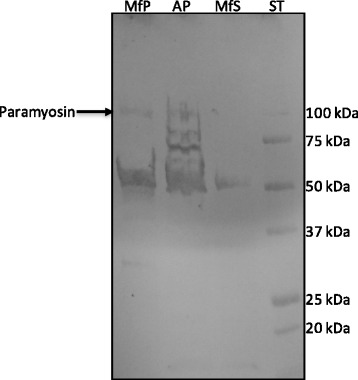
Western blot of native paramyosin. Native paramyosin extracted from microfilariae and adults was detected by serum (1:1000) from dogs with confirmed *Onchocerca lupi* infections. *Abbreviations*: MfP, microfilarial protein pellet; AP, adult protein; MfS, microfilarial protein supernatant

Western blot analysis revealed that serum from dogs infected with *O. lupi* readily detected native paramyosin purified from microfilariae (< 0.5 ng, lower than the detection limit of the Qubit® Protein Assay Kit) and adults (< 1 μg) (Fig. 5). An additional co-purifying protein (~50 kDa) was detected, but was also detected by negative serum (data not shown). The microfilarial protein was detected, whilst the western blot of adults contained many additional proteins, most likely from the dog, as the adults are often embedded within the eye tissue, which cannot be completely removed. As DNA, RNA and protein are extracted from each sample simultaneously, enzyme digestion to facilitate removal of dog tissue was not performed due to the concurrent risk of RNA degradation. None of the purified, Factor Xa cleaved peptides, which were of the expected sizes, were detected by serum from dogs with *O. lupi*.

## Discussion

*Onchocerca lupi* paramyosin (*Ol-para*, 2,643 bp cDNA) was herein isolated and characterised in adults, both males and females, and microfilariae. The predicted *Ol-*PARA protein (i.e. 881 amino acids, 101.24 kDa) represents a unique set of data for filarioids nematodes and, most specifically, for this little known vector-borne helminth. Indeed, although *O. lupi* is known to cause morbidity in both humans and animals, very little molecular information is available. The length of *Ol-para* is consistent with those of other filarioid nematodes, which range from the smallest 2,508 bp (*L. loa*, NCBI Accession no. XM_003141046.1) to 2,640 bp (*B. malayi*, NCBI Accession no. Q01202.2). The nucleotide identity levels also reflect the relatedness of *Ol*-*para* compared with paramyosin sequences from other organisms, with high homology to other filarioid nematodes (91–98 %) compared with non-filarioid nematodes (~77 %) and the platyhelminths (~52 %). The AT-richness of *Ol-para* (i.e. 60.23 %) was consistent with other filarioid nematode sequences, including *O. volvulus* (AT 61 %), *L. loa* (AT 61 %) and *B. malayi* (AT 61 %). Some, but not all, of the platyhelminth paramyosins (*Schistosoma* spp.) are also AT-rich (65–67 %), whilst the remaining platyhelminths and non-filarioid nematodes have percentages of AT nucleotides in their sequences of between 44 % and 54 % (Table 2). Whether there is any functional significance of this difference in AT proportions in paramyosin molecules is yet to be determined.

Unlike some other species of nematodes, however, only one transcript encoding paramyosin was detected in *O. lupi* adults and microfilariae, whilst no paramyosin transcript was detected in eggs. The detection of transcription of paramyosin in *O. lupi* microfilariae is consistent with what has been found in other nematodes. Indeed, two forms of paramyosin have been detected in *B. malayi* (NCBI Accession no. P13392.2), which differ in size by only three base pairs from adults (NCBI Accession no. P13392). Paramyosin transcripts in other species, such as the free-living nematode *Caenorhabditis elegans*, differ significantly in size (WormBase Accession no. F07A5.7a and b, 2,616 and 1,659 bp, respectively). In *O. lupi* it is possible that additional transcripts exist, but may be expressed at levels not able to be detected by the methods used. Upon deep transcriptomic sequencing of *B. malayi* life-cycle stages, paramyosin and ELF1α were identified across the life-cycle of the parasite [59]. Similarly, in *Dictyocaulus viviparus* (cattle lungworm) and *Clonorchis sinensis* (liver fluke) paramyosin is expressed throughout the life-cycle [60, 61]. Undoubtedly, next generation sequencing of the *O. lupi* transcriptome could be useful in determining the number of transcripts and confirming the expression patterns of paramyosin across the parasite life-cycle.

The *Ol*-PARA sequence is also consistent with that of other filarioid nematodes, with highest amino acid identity with the *O. volvulus, B. malayi* and *L. loa*, and within the range of lengths of proteins (90–93 % identity, 836–880 amino acids). Like other filarioid proteins, it has no signal peptides or transmembrane spanning domains. The *Ol*-PARA sequence, like five of 11 sequences available for other nematode species (*A. caninum*, *B. malayi, C. elegans*, *D. viviparus* and *O. volvulus*), has a proton donor site. This motif is commonly associated with enzyme catalysis of hydrolysis of 1,3- or 1,4- bonds in β-D-glucans (prosite.expasy.org), but the functional significance of this activity in paramyosin is not yet known and is, as yet, unreported in the available literature. There is evidence from some nematode species to suggest that there is a short and a long isoform of paramyosin, hence the differences in size (Table 3). However, there is not enough information available for many species to confirm this. Phylogenetic analysis of platyhelminth and nematode proteins indicated that, whilst these proteins share conserved T- and B-cell epitopes, they are distinctly different proteins. Based on the phylogenesis of paramyosin molecules, five groups of nematodes were recognized: the rhabditids, including *Caenorhabditis* spp., the strongylids (*Haemonchus contortus* and *A. caninum*), the metastrongylids (*Dictyocaulus viviparous*)*,* the ascarids (*Anisakis* spp. and *Ascaris suum*) and the filarioids (*Onchocherca* spp., *B. malayi*, *A. viteae*, *L. loa* and *D. immitis*). The *W. bancrofti* paramyosin does not closely cluster with other filarioids (Fig. 4). It is possible that for *W. bancrofti* that only one of a number of transcripts has been identified.

Proteomic analysis of *Ol*-PARA has indicated that this protein, like other paramyosins, has the potential to elicit both humoral and cellular immune responses in the host [31]. The bioinformatic approach in this study, along with recombinant peptide expression, was however unable to identify the antibody binding epitope(s) of this protein. Nonetheless, 28 published epitopes (14 T- and 14 B-cell) experimentally determined in platyhelminths, ticks and *O. volvulus*, were conserved in *Ol*-PARA [38–43, 62–67].

In addition, paramyosin is a vaccine candidate, predominantly in platyhelminths and also in *Trichinella spiralis* [41], and it has been listed by the World Health Organization as one of six antigen candidates for vaccines against *Schistosoma* spp. [64]. This, including the positive antibody binding of very small amounts of microfilarial and adult native paramyosin detected herein, suggests that *Ol*-PARA should be tested as a potential vaccine candidate. Accordingly, *Ol*-*para* could also be tested as a DNA vaccine, particularly given the greater ease with which DNA can be manipulated when compared with proteins. To date, four studies have assessed paramyosin DNA-based vaccination (100 μg per rat/mouse) against *Schistosoma japonicum* [68, 69], *B. malayi* [70] and *T. solium* [71], with evidence of some protective effect. Nonetheless, for its high antigenicity, *Ol*-PARA could also be exploited for the development of a serological diagnostic test. Indeed, this antigen (Og4C3) has been used for the preparation of a number of diagnostic kits based on the detection of *O. gibsoni* paramyosin in cattle, which may also cross-react with filarioids infecting humans such as *W. bancrofti* and *B. malayi* [27–29, 32] and, to some extent, with *O. lupi* in serum from dogs that scored positive for microfilariae [30]. In addition, the identification of 621 antibody binding epitopes, unique to *O. lupi* when compared with other filarioid nematodes, provides a dataset invaluable for the development of a serological diagnostic assay through specific targeting of one or more of these epitopes. Antibody detection of *O. lupi* would not only assist in the detection of subclinically infected animals, gaining a better understanding of the true distribution and prevalence of *O. lupi*, but would also be amenable to the rapid screening of potential insect vectors. Vaccine studies would determine whether this molecule can provide a protective effect against infection by a zoonotic nematode for which the full zoonotic potential is yet to be determined. Future work will involve recombinant expression (eukaryotic) of the full protein, production of antibodies and testing of the serum against a range of filarial and other nematodes infecting humans and animals to ensure specificity. Serum from animals with infections of other filarioid nematodes will also be tested against the recombinant protein. If there is no cross-reactivity with serum from animals with other filarioid nematode infections this would provide a strong basis for the development of a specific serological diagnostic test.

## Conclusions

This study indicates that paramyosin is a suitable candidate for the development of a specific diagnostic test and as a potential vaccine candidate, avoiding in the future a need for invasive, time-consuming tests. The development of a specific diagnostic test will enable further studies on the distribution and prevalence of this little known zoonotic filarioid.

## Additional file

Additional file 1: Figure S1. Epitopes predicted by bioinformatics and those reported in the literature for other organisms mapped to *Onchocerca lupi* paramyosin. Residues underlined indicate T-cell epitopes whilst those in bold indicate B-cell epitopes. Of the 874 predicted antibody epitopes, when compared with those predicted for *Acanthocheilonema viteae*, *Brugia malayi*, *Dirofilaria immitis*, *Loa loa*, *Onchocerca volvulus* and *Wuchereria bancrofti*, 621 were unique to *Onchocerca lupi*. (DOCX 32 kb)
