# Supplementary material for: Paramyosin of canine Onchocerca lupi: usefulness for the diagnosis of a neglected zoonotic disease
Source: Parasit Vectors. 2016 Sep 7;9(1):493. doi: 10.1186/s13071-016-1783-z (PMC5013582; doi:10.1186/s13071-016-1783-z)
Supplement: Additional file 1: Figure S1. — Epitopes predicted by bioinformatics and those reported in the literature for other organisms mapped to Onchocerca lupi paramyosin. Residues underlined indicate T-cell epitopes whilst those in bold indicate B-cell epitopes. Of the 874 predicted antibody epitopes, when compared with those predicted for Acanthocheilonema viteae, Brugia malayi, Dirofilaria immitis, Loa loa, Onchocerca volvulus and Wuchereria bancrofti, 621 were unique to Onchocerca lupi. (DOCX 32 kb) [file 13071_2016_1783_MOESM1_ESM.docx]

Figure S1: Epitopes predicted by bioinformatics and those reported in the literature for other organisms mapped to *Onchocerca lupi* paramyosin. Residues underlined indicate T-cell epitopes whilst those in bold indicate B-cell epitopes. Of the 874 predicted antibody epitopes, when compared with those predicted for *Acanthocheilonema viteae, Brugia malayi, Dirofilaria immitis, Loa loa, Onchocerca volvulus* and *Wuchereria bancrofti*, 621 were unique to *Onchocerca lupi*.

O_lupi MSGSLYRSPSAALYKSPSMSAFGGLPAAFGSMSVADLGS**LTLL**KDKIRLLQEDLESEREL

67 -------------------------------MSVADLG----------------------

71 --------------------------------------------------------EREL

81 -------------YKSPSMS----------------------------------------

121 ---------------------------------------LTLLKDK--------------

181 -----------------SMSAFGG------------------------------------

205 -------------------SAFGGLP----------------------------------

265 --------------KSPSMSA---------------------------------------

287 --------PSAALYK---------------------------------------------

305 -----------------------------GSMSVAD------------------------

353 ------------------------------------LGSLTLL-----------------

393 ----------------------------------------------------------EL

397 --------------------AFGGLPA---------------------------------

431 ----------------------------FGSMSVA-------------------------

537 --------------------------------SVADLGS---------------------

579 -------------------------------------------------------SEREL

583 -------------------------------------GSLTLLK----------------

653 -----------------------------------------------------LESEREL

679 -------SPSAALY----------------------------------------------

715 -----------------------------------------------RLLQEDL------

755 -----------------------------------DLGSLTL------------------

769 -----------------------------------------------------------L

787 ------------------------------------------------LLQEDLE-----

805 ---------------------------------------------------EDLESER--

837 ----LYRSPSA-------------------------------------------------

845 ------------------------------SMSVADL-----------------------

857 ----------------------GGLPAAF-------------------------------

869 -----YRSPSAA------------------------------------------------

917 -----------ALYKSPS------------------------------------------

955 ----------------------------------------------IRLLQED-------

957 ------------------------LPAAFGS-----------------------------

991 --------------------------------------SLTLLKD---------------

1011 ------------------------------------------------------ESEREL

1021 -----------------------GLPAAFG------------------------------

1045 ---------------------------AFGSMSV--------------------------

1055 ---------------------------------------------------------REL

1065 ---------SAALYKS--------------------------------------------

1073 -------------------------PAAFGSM----------------------------

1079 ----------------PSMSAFG-------------------------------------

1105 -SGSLYRS----------------------------------------------------

1111 ------RSPSAAL-----------------------------------------------

1117 ------------------------------------------LKDKIRL-----------

1131 -----------------------------------------LLKDKIR------------

1179 ---------------------------------VADLGSL--------------------

1231 ----------AALYKSP-------------------------------------------

1237 ----------------------------------ADLGSLT-------------------

1251 --------------------------------------------DKIRLLQ---------

1257 --GSLYRSP---------------------------------------------------

1307 ----------------------------------------------------DLESERE-

1329 -------------------------------------------KDKIRLL----------

1363 --------------------------AAFGSMS---------------------------

1383 ---------------SPSMSAF--------------------------------------

1433 ---------------------FGGLPAA--------------------------------

1453 ------------------MSAFGGL-----------------------------------

1467 -------------------------------------------------LQEDLES----

1481 ----------------------------------------TLLKDKI-------------

1503 --------------------------------------------------QEDLESE---

1533 MSGSLYR-----------------------------------------------------

1557 ---SLYRSPS--------------------------------------------------

1585 ------------LYKSPSM-----------------------------------------

O_lupi RNRIERERADLSVQLIALTDRLEDAEGTTDSQIESNRKREAELQKLRK**LLEESQ**LENEDA

27 -----------SVQLIAL------------------------------------------

71 RNR---------------------------------------------------------

87 --------------LIALTDR---------------------------------------

119 ----------------------------------------AELQKLR-------------

133 --------------------------------------------KLRKLLE---------

137 ----------------------------------------------RKLLEES-------

145 -----------------------------------------ELQKLRK------------

185 ---------------------------------------------------------EDA

227 -------------------------------------------------------ENEDA

255 -----RERADLS------------------------------------------------

261 -------------------------------------KREAELQ----------------

291 ---IERERAD--------------------------------------------------

319 ----------------------------TDSQIES-------------------------

321 ----------------------------------------------------------DA

335 ----------LSVQLIA-------------------------------------------

357 --------ADLSVQL---------------------------------------------

365 -------------------------EGTTDSQ----------------------------

393 RNRIE-------------------------------------------------------

413 ------------------------------------------------LLEESQL-----

433 ----------------ALTDRLE-------------------------------------

477 -----------------------------------------------KLLEESQ------

555 -NRIERER----------------------------------------------------

579 RN----------------------------------------------------------

607 -----------------------------------NRKREAE------------------

619 ------------------------------------RKREAEL-----------------

651 -------------QLIALTD----------------------------------------

699 -----------------LTDRLED------------------------------------

719 ---------------------------------ESNRKRE--------------------

769 RNRIER------------------------------------------------------

777 --------------------------------------------------------NEDA

797 ------------------------------------------------------LENEDA

829 -----------------------------------------------------QLENEDA

853 ---------------------------------------------LRKLLEE--------

881 -------------------------------------------------LEESQLE----

899 ------------------------AEGTTDS-----------------------------

907 ------ERADLSV-----------------------------------------------

921 --RIERERA---------------------------------------------------

995 --------------------------GTTDSQI---------------------------

1011 R-----------------------------------------------------------

1055 RNRI--------------------------------------------------------

1101 -----------------------DAEGTTD------------------------------

1125 -------RADLSVQ----------------------------------------------

1149 -----------------------------DSQIESN------------------------

1163 ---------------IALTDRL--------------------------------------

1205 ---------------------------------------------------ESQLENE--

1207 ------------------TDRLEDA-----------------------------------

1221 ----------------------------------SNRKREA-------------------

1235 --------------------RLEDAEG---------------------------------

1353 ------------------------------------------LQKLRKL-----------

1357 RNRIERE-----------------------------------------------------

1361 ----------------------------------------------------SQLENED-

1367 ---------------------LEDAEGT--------------------------------

1371 ---------------------------TTDSQIE--------------------------

1377 -------------------------------QIESNRK----------------------

1409 ---------DLSVQLI--------------------------------------------

1415 ----ERERADL-------------------------------------------------

1451 ----------------------EDAEGTT-------------------------------

1461 --------------------------------------REAELQK---------------

1483 ------------------------------SQIESNR-----------------------

1501 -------------------DRLEDAE----------------------------------

1507 -----------------------------------------------------------A

1515 -------------------------------------------QKLRKLL----------

1603 --------------------------------IESNRKR---------------------

1707 ------------VQLIALT-----------------------------------------

1711 ---------------------------------------EAELQKL--------------

1719 --------------------------------------------------EESQLEN---

O_lupi MNVLRKKHQDACLDYTEQIEQLQKKNSKIDRERQRLQHEVIELTATIDQLQKDKHLAEKA

5 ------------------------KNSKIDR-----------------------------

21 --------------------------------------------------QKDKHLA---

103 -----------------------------------------------------------A

107 ------------------------------------------LTATIDQ-----------

151 -------------------------------------------------------LAEKA

185 MNVL--------------------------------------------------------

227 MN----------------------------------------------------------

239 -------------------------------ERQRLQH----------------------

245 MNVLRKK-----------------------------------------------------

299 -------------------------------------------TATIDQL----------

313 ------KHQDACL-----------------------------------------------

321 MNVLR-------------------------------------------------------

341 ------------------------------------QHEVIEL-----------------

369 ----------------------------IDRERQR-------------------------

419 ----RKKHQDA-------------------------------------------------

443 ----------------------------------------------------DKHLAEK-

445 ------------------------------RERQRLQ-----------------------

467 -----------------------------------------------------KHLAEKA

469 -----------------------------------------ELTATID------------

505 -------------------------NSKIDRE----------------------------

527 --VLRKKHQ---------------------------------------------------

533 --------QDACLDY---------------------------------------------

547 -------------------------------------------------LQKDKHL----

587 ---------------------------KIDRERQ--------------------------

677 ------------------------------------------------QLQKDKH-----

731 --------------------------------RQRLQHE---------------------

733 -----------------------KKNSKID------------------------------

737 ---LRKKHQD--------------------------------------------------

777 MNV---------------------------------------------------------

795 ------------------------------------------------------HLAEKA

797 M-----------------------------------------------------------

819 ---------------TEQIEQL--------------------------------------

849 ----------------------------------RLQHEVI-------------------

871 ---------DACLDYT--------------------------------------------

903 ---------------------------------------VIELTAT--------------

925 -----------------------------DRERQRL------------------------

975 ----------------------------------------------IDQLQKD-------

1005 ------------------IEQLQKK-----------------------------------

1009 -------------DYTEQIE----------------------------------------

1031 ------------LDYTEQI-----------------------------------------

1051 --------------------QLQKKNS---------------------------------

1067 ----------ACLDYTE-------------------------------------------

1085 ----------------------------------------------------------KA

1141 -----KKHQDAC------------------------------------------------

1151 -----------------------------------LQHEVIE------------------

1169 ---------------------LQKKNSK--------------------------------

1203 -------------------EQLQKKN----------------------------------

1239 --------------------------------------------------------AEKA

1303 -----------------------------------------------DQLQKDK------

1385 --------------YTEQIEQ---------------------------------------

1425 -----------------QIEQLQK------------------------------------

1489 ----------------------------------------IELTATI-------------

1507 MNVLRK------------------------------------------------------

1579 ---------------------------------------------TIDQLQK--------

1591 -------------------------------------HEVIELT----------------

1593 ----------------------QKKNSKI-------------------------------

1629 ---------------------------------------------------KDKHLAE--

1631 -------HQDACLD----------------------------------------------

1649 --------------------------------------EVIELTA---------------

1655 ----------------EQIEQLQ-------------------------------------

1669 --------------------------SKIDRER---------------------------

1705 -NVLRKKH----------------------------------------------------

1713 ---------------------------------------------------------EKA

1717 --------------------------------------------ATIDQLQ---------

1727 ---------------------------------QRLQHEV--------------------

O_lupi AERFEAQTIELSNKVEDLNRTCQRFSTTTSTFTSRNNDLLKEIHDQKVQLDNLQHVKYQL

43 --------------------------------TSRNNDL---------------------

53 -----------------------------------------------------------L

63 -------TIELSNK----------------------------------------------

79 ------------------------------TFTSRNN-----------------------

83 -----------------LNRTCQR------------------------------------

103 AERFEA------------------------------------------------------

123 ---------------------------------------------------NLQHVKY--

129 ----------------------------------RNNDLLK-------------------

151 AE----------------------------------------------------------

165 --------------------TCQRFST---------------------------------

215 -------------------------------------------HDQKVQL----------

231 -------------------------------------------------LDNLQHV----

237 ------------------NRTCQRF-----------------------------------

241 --------------VEDLNRT---------------------------------------

243 ----------------------------TSTFTSR-------------------------

259 --------------------------------------------------DNLQHVK---

311 ----------------------------------------------------------QL

315 --------IELSNKV---------------------------------------------

337 ---------------------------TTSTFTS--------------------------

405 ----------LSNKVED-------------------------------------------

457 --RFEAQTI---------------------------------------------------

545 ----------------------QRFSTTT-------------------------------

581 -----------------------RFSTTTS------------------------------

611 ----------------------------------------KEIHDQK-------------

633 ---------------------------------------------QKVQLDN--------

655 AERFEAQ-----------------------------------------------------

745 ---------------EDLNRTC--------------------------------------

781 --------------------------------------------DQKVQLD---------

795 A-----------------------------------------------------------

817 ----------------DLNRTCQ-------------------------------------

827 -------------------------------FTSRNND----------------------

835 ---FEAQTIE--------------------------------------------------

877 -ERFEAQT----------------------------------------------------

889 --------------------------TTTSTFT---------------------------

897 ------------------------------------------------------HVKYQL

911 ------------------------------------NDLLKEI-----------------

949 ---------------------CQRFSTT--------------------------------

983 -------------------------------------------------------VKYQL

997 ------------------------------------------IHDQKVQ-----------

1033 -----------SNKVEDL------------------------------------------

1049 -------------KVEDLNR----------------------------------------

1085 AERFE-------------------------------------------------------

1133 ---------------------------------------LKEIHDQ--------------

1181 ------------------------------------------------QLDNLQH-----

1189 --------------------------------------LLKEIHD---------------

1239 AER---------------------------------------------------------

1253 -----------------------------------------------VQLDNLQ------

1263 -----------------------------STFTSRN------------------------

1265 ----------------------------------------------KVQLDNL-------

1395 ---------ELSNKVE--------------------------------------------

1459 -------------------RTCQRFS----------------------------------

1475 -------------------------STTTSTF----------------------------

1491 -----------------------------------NNDLLKE------------------

1527 ----------------------------------------------------LQHVKYQ-

1539 ----EAQTIEL-------------------------------------------------

1559 ---------------------------------SRNNDLL--------------------

1565 -----AQTIELS------------------------------------------------

1571 ------QTIELSN-----------------------------------------------

1575 -----------------------------------------------------QHVKYQL

1597 ------------NKVEDLN-----------------------------------------

1651 -----------------------------------------EIHDQKV------------

1671 ------------------------FSTTTST-----------------------------

1675 --------------------------------------------------------KYQL

1693 -------------------------------------DLLKEIH----------------

1699 ---------------------------------------------------------YQL

1713 AERF--------------------------------------------------------

O_lupi AQQLEEARRRLEDAERERSQLQAQLHQVQLELDSVRTALDE**ESAARAE**AEHKLALGQIPK

53 AQQLEE------------------------------------------------------

61 ---------------------------------------------RAEAEHK--------

99 ---------------------------------------DEESAAR--------------

131 -----------------------------------RTALDEE------------------

141 ----EEARRRL-------------------------------------------------

177 --------------------------------------------ARAEAEH---------

183 ------------------------------ELDSVRT-----------------------

195 --------------------------------------------------------QIPK

199 -----------------RSQLQAQ------------------------------------

225 ---------RLEDAER--------------------------------------------

235 -------------------------------------------------EHKLALG----

307 ------------------------------------TALDEES-----------------

311 AQQLE-------------------------------------------------------

327 --------------------------------DSVRTAL---------------------

329 -------RRRLEDA----------------------------------------------

351 ---------------------------------------------------------IPK

385 --------------ERERSQL---------------------------------------

447 -------------------------HQVQLEL----------------------------

455 -------------AERERSQ----------------------------------------

503 ---------------------------------SVRTALD--------------------

521 -------------------QLQAQLH----------------------------------

523 ------------------------------------------------AEHKLAL-----

531 ---------------------------VQLELDS--------------------------

549 -----------------------------------------------EAEHKLA------

575 --QLEEARR---------------------------------------------------

615 ------------------------LHQVQLE-----------------------------

631 ----------------------------------VRTALDE-------------------

639 ----------------------------------------------------------PK

753 -------------------------------------------------------GQIPK

779 AQQLEEA-----------------------------------------------------

825 -------------------------------LDSVRTA----------------------

833 ------------------------------------------------------LGQIPK

859 -----EARRRLE------------------------------------------------

897 A-----------------------------------------------------------

919 -------------------------------------------AARAEAE----------

933 -----------EDAERER------------------------------------------

945 ----------------------------QLELDSV-------------------------

951 --------------------------------------------------HKLALGQ---

983 AQ----------------------------------------------------------

987 ----------LEDAERE-------------------------------------------

989 ---------------------------------------------------KLALGQI--

1113 ------------DAERERS-----------------------------------------

1135 --------------------------QVQLELD---------------------------

1167 --------------------------------------LDEESAA---------------

1173 ----------------------AQLHQVQ-------------------------------

1195 ----------------------------------------------AEAEHKL-------

1223 ---------------RERSQLQ--------------------------------------

1255 ------ARRRLED-----------------------------------------------

1267 ----------------ERSQLQA-------------------------------------

1269 -----------------------------LELDSVR------------------------

1273 --------RRLEDAE---------------------------------------------

1275 -----------------------QLHQVQL------------------------------

1309 ------------------SQLQAQL-----------------------------------

1339 -QQLEEAR----------------------------------------------------

1351 ----------------------------------------EESAARA-------------

1427 ----------------------------------------------------LALGQIP-

1441 --------------------LQAQLHQ---------------------------------

1455 -----------------------------------------ESAARAE------------

1499 ---LEEARRR--------------------------------------------------

1529 ---------------------QAQLHQV--------------------------------

1617 -------------------------------------ALDEESA----------------

1623 ------------------------------------------SAARAEA-----------

1641 -----------------------------------------------------ALGQIPK

1663 -----------------------------------------------------------K

1675 AQQ---------------------------------------------------------

1699 AQQL--------------------------------------------------------

O_lupi LPNGRSKFDAEVLFIMKKWKIYERKCCKSQLNWRNNW**KYMLQKI**SQLEKAKSRLQSEVEV

23 --------------------------------------YMLQKIS---------------

41 -------------FIMKKWK----------------------------------------

59 -----SKFDAEV------------------------------------------------

113 ----------------------------------------------LEKAKSR-------

171 -----------------------------QLNWRNN------------------------

191 ---------------------------------------------------------VEV

195 LPN---------------------------------------------------------

281 ------------------------KCCKSQL-----------------------------

293 -----------------------------------------------EKAKSRL------

295 -------------------------CCKSQLN----------------------------

351 LPNG--------------------------------------------------------

371 --------------------------------------------SQLEKAK---------

379 -------------------------------------------------------SEVEV

383 --------------------------CKSQLNW---------------------------

391 -----------------KWKIYER------------------------------------

403 ----------------KKWKIYE-------------------------------------

427 -----------VLFIMKK------------------------------------------

473 --------------------IYERKCC---------------------------------

487 -------FDAEVLF----------------------------------------------

493 ----------------------------------------------------RLQSEVE-

519 -PNGRSKF----------------------------------------------------

525 ------------------------------------------------------QSEVEV

565 --------DAEVLFI---------------------------------------------

571 ---------------MKKWKIY--------------------------------------

603 -----------------------------------------------------LQSEVEV

609 ---------------------------KSQLNWR--------------------------

639 LPNGR-------------------------------------------------------

645 ----------------------------------------LQKISQL-------------

665 -----------------------------------------QKISQLE------------

691 ---------------------------------------------QLEKAKS--------

725 --------------IMKKWKI---------------------------------------

753 LP----------------------------------------------------------

793 --------------------------------------------------KSRLQSE---

807 ------------------------------LNWRNNW-----------------------

861 ------------------------------------------KISQLEK-----------

867 --------------------------------WRNNWKY---------------------

927 ----RSKFDAE-------------------------------------------------

953 -----------------------------------------------------------V

1027 ---------------------------------------------------SRLQSEV--

1071 -----------------------------------NWKYMLQ------------------

1099 -------------------------------------KYMLQKI----------------

1127 -----------------------RKCCKSQ------------------------------

1165 ------------------WKIYERK-----------------------------------

1197 ------KFDAEVL-----------------------------------------------

1261 ------------LFIMKKW-----------------------------------------

1285 --------------------------------------------------------EVEV

1311 -------------------------------NWRNNWK----------------------

1317 --NGRSKFD---------------------------------------------------

1331 LPNGRSK-----------------------------------------------------

1335 ---------AEVLFIM--------------------------------------------

1341 ---------------------YERKCCK--------------------------------

1355 ------------------------------------WKYMLQK-----------------

1369 ---GRSKFDA--------------------------------------------------

1379 ------------------------------------------------KAKSRLQ-----

1485 -------------------------------------------ISQLEKA----------

1531 ----------EVLFIMK-------------------------------------------

1583 ----------------------------SQLNWRN-------------------------

1633 ---------------------------------------MLQKISQ--------------

1645 -------------------KIYERKC----------------------------------

1663 LPNGRS------------------------------------------------------

1685 ----------------------------------------------------------EV

1691 ----------------------------------NNWKYML-------------------

1715 ----------------------ERKCCKS-------------------------------

1739 -------------------------------------------------AKSRLQS----

O_lupi L**IVDLEKAQNTIAI**LER**AKEQLE**KTVNELKVRIDELTVELEAAQR**EARAA**LAELQKMKNL

3 ----------------------------------------EAAQREA-------------

9 ------------------------------------------------AALAELQ-----

51 ---------------------------------DELTVEL--------------------

57 --------------------------------------------REARAAL---------

73 ----------TIAILER-------------------------------------------

85 ---------------ERAKEQL--------------------------------------

143 ----------------RAKEQLE-------------------------------------

191 LIVD--------------------------------------------------------

251 ---------NTIAILE--------------------------------------------

271 -----------------------------KVRIDEL------------------------

273 -----------IAILERA------------------------------------------

325 --------------------QLEKTVN---------------------------------

379 LI----------------------------------------------------------

395 --------------------------------------------------------MKNL

441 -------------------------------------VELEAAQ----------------

449 ----------------------------------------------------------NL

511 --------------LERAKEQ---------------------------------------

513 ----------------------------LKVRIDE-------------------------

515 ------------AILERAK-----------------------------------------

525 L-----------------------------------------------------------

595 -------------------------------------------QREARAA----------

613 -----------------------------------------------------LQKMKNL

637 ---------------------------------------LEAAQRE--------------

641 ----------------------EKTVNEL-------------------------------

689 -----------------AKEQLEK------------------------------------

703 -------AQNTIAI----------------------------------------------

717 -------------------------VNELKVR----------------------------

741 -------------ILERAKE----------------------------------------

751 -----------------------------------LTVELEA------------------

761 --------------------------NELKVRI---------------------------

771 -----------------------------------------------RAALAEL------

775 -----------------------------------------AAQREAR------------

785 -------------------------------RIDELTV----------------------

875 ------------------------------------------AQREARA-----------

953 LIVDLE------------------------------------------------------

965 ----------------------------------ELTVELE-------------------

1019 -------------------------------------------------ALAELQK----

1041 ------------------------------------TVELEAA-----------------

1069 ------------------KEQLEKT-----------------------------------

1175 ----LEKAQNT-------------------------------------------------

1185 --------------------------------IDELTVE---------------------

1193 ---------------------------------------------EARAALA--------

1215 ---------------------------ELKVRID--------------------------

1277 ---------------------------------------------------------KNL

1279 ------------------------------------------------------QKMKNL

1285 LIV---------------------------------------------------------

1289 LIVDLEK-----------------------------------------------------

1301 -----EKAQNTI------------------------------------------------

1343 ----------------------------------------------------ELQKMKN-

1373 -----------------------KTVNELK------------------------------

1387 ----------------------------------------------ARAALAE-------

1391 --VDLEKAQ---------------------------------------------------

1411 --------------------------------------ELEAAQR---------------

1437 --------------------------------------------------LAELQKM---

1447 ---DLEKAQN--------------------------------------------------

1511 ---------------------------------------------------AELQKMK--

1519 -------------------------------------------------------KMKNL

1613 --------QNTIAIL---------------------------------------------

1627 ------------------------------VRIDELT-----------------------

1665 -IVDLEKA----------------------------------------------------

1679 ------------------------TVNELKV-----------------------------

1685 LIVDL-------------------------------------------------------

1687 ---------------------LEKTVNE--------------------------------

1729 -------------------EQLEKTV----------------------------------

1731 ------------------------------------------------------------

1733 ------------------------------------------------------------

1735 ------------------------------------------------------------

1737 ------KAQNTIA-----------------------------------------------

1739 ------------------------------------------------------------

1741 ------------------------------------------------------------

1743 ------------------------------------------------------------

1745 ------------------------------------------------------------

O_lupi YEKAVEQKEALARENKKLQDDLHEAKEALADANRKLHELDLENAR**LAGEIREL**QTALKES

49 --------------------DLHEAKE---------------------------------

55 -------------ENKKLQD----------------------------------------

95 -----------------------------------------------------QTALKES

105 -EKAVEQK----------------------------------------------------

117 -------------------DDLHEAK----------------------------------

125 ----------------------------------------------AGEIREL-------

153 ---------------------------------RKLHELD--------------------

161 ----------------------------------KLHELDL-------------------

163 -----------------LQDDLHE------------------------------------

175 ---------------------------ALADANR--------------------------

189 --------------------------EALADAN---------------------------

207 -----------ARENKKL------------------------------------------

223 ----------LARENKK-------------------------------------------

285 ----------------KLQDDLH-------------------------------------

303 ------------------------------------------------------TALKES

349 --------------------------------------------------RELQTAL---

377 --------------------------------------------RLAGEIR---------

395 YEK---------------------------------------------------------

411 YEKAVE------------------------------------------------------

415 -------------------------------ANRKLHE----------------------

425 ------------------------AKEALAD-----------------------------

439 ---------------KKLQDDL--------------------------------------

449 YEKAV-------------------------------------------------------

501 -----------------------------------LHELDLE------------------

541 ----------------------------------------LENARLA-------------

675 ----VEQKEAL-------------------------------------------------

683 ---AVEQKEA--------------------------------------------------

685 --------EALAREN---------------------------------------------

687 ------QKEALAR-----------------------------------------------

697 -----------------------------------------------------------S

735 -------------------------------------------ARLAGEI----------

739 --------------------------------------LDLENAR---------------

749 --------------NKKLQDD---------------------------------------

799 ------------------------------------------NARLAGE-----------

803 -----------------------------ADANRKL------------------------

809 ------------------QDDLHEA-----------------------------------

813 --KAVEQKE---------------------------------------------------

891 ---------------------------------------------------ELQTALK--

893 -------------------------KEALADA----------------------------

913 ----------------------HEAKEAL-------------------------------

915 -----------------------------------------ENARLAG------------

1053 ---------------------------------------------LAGEIRE--------

1075 -------KEALARE----------------------------------------------

1081 -----EQKEALA------------------------------------------------

1095 -------------------------------------------------------ALKES

1097 --------------------------------------------------------LKES

1153 -------------------------------------ELDLENA----------------

1155 -----------------------EAKEALA------------------------------

1243 --------------------------------NRKLHEL---------------------

1277 YEKA--------------------------------------------------------

1279 Y-----------------------------------------------------------

1359 ------------------------------DANRKLH-----------------------

1389 ---------ALARENK--------------------------------------------

1439 ----------------------------LADANRK-------------------------

1517 ---------------------LHEAKEA--------------------------------

1519 YE----------------------------------------------------------

1541 -----------------------------------------------GEIRELQ------

1567 ----------------------------------------------------------ES

1573 YEKAVEQ-----------------------------------------------------

1581 ------------------------------------HELDLEN-----------------

1643 ---------------------------------------------------------KES

1659 ------------RENKKLQ-----------------------------------------

1667 -------------------------------------------------IRELQTA----

1677 ------------------------------------------------EIRELQT-----

1701 ----------------------------------------------------LQTALKE-

1723 ---------------------------------------DLENARL--------------

O_lupi E**AARRDAENRAQRAL**AELQQLRIEMER**RLQEKEEEM**EALRKNMQFEIDRLTAALADAEAR

11 ----------------------IEMERRL-------------------------------

25 -----------------------EMERRLQ------------------------------

37 -AARRDAE----------------------------------------------------

109 ----------------ELQQLRI-------------------------------------

127 ----------------------------LQEKEEE-------------------------

179 ----------------------------------------------------ALADAEA-

197 --------------------------------EEEMEAL---------------------

247 ---------------------------------------RKNMQFE--------------

249 --------------------------------------LRKNMQF---------------

275 ----------------------------------EMEALRK-------------------

333 ----RDAENRA-------------------------------------------------

339 ---------------------------------------------EIDRLTA--------

363 ---RRDAENR--------------------------------------------------

461 -------------------------------------------------------DAEAR

489 EAARRDA-----------------------------------------------------

491 ----------------------------------------------------------AR

495 --------------------------------------------------------AEAR

517 -------------------------------------------------LTAALAD----

535 --------------------------------------------------TAALADA---

543 --------------------------------------------FEIDRLT---------

577 -------------------------ERRLQEK----------------------------

629 ---------------------------RLQEKEE--------------------------

647 -------------------------------KEEEMEA----------------------

663 -----------------------------------------------DRLTAAL------

697 EAARRD------------------------------------------------------

721 --------------LAELQQL---------------------------------------

757 ---------------AELQQLR--------------------------------------

759 -------------ALAELQQ----------------------------------------

841 --------NRAQRAL---------------------------------------------

843 ---------------------------------------------------------EAR

865 ---------RAQRALA--------------------------------------------

873 --------------------LRIEMER---------------------------------

885 ------------------------------------EALRKNM-----------------

935 ------------------------------------------------------ADAEAR

971 -----DAENRAQ------------------------------------------------

1007 -------ENRAQRA----------------------------------------------

1023 ------------------------------EKEEEME-----------------------

1047 -----------------------------QEKEEEM------------------------

1057 -----------QRALAEL------------------------------------------

1095 EA----------------------------------------------------------

1097 EAA---------------------------------------------------------

1119 ----------------------------------------KNMQFEI-------------

1129 -------------------QLRIEME----------------------------------

1145 -----------------------------------MEALRKN------------------

1171 --ARRDAEN---------------------------------------------------

1227 -----------------------------------------------------------R

1245 -------------------------------------ALRKNMQ----------------

1283 ------------------------------------------------RLTAALA-----

1393 ---------------------------------EEMEALR--------------------

1417 -------------------------------------------QFEIDRL----------

1429 -----------------------------------------------------LADAEAR

1449 ---------------------------------------------------AALADAE--

1473 ----------AQRALAE-------------------------------------------

1509 -----------------LQQLRIE------------------------------------

1535 ------------RALAELQ-----------------------------------------

1543 ------------------------MERRLQE-----------------------------

1553 ------------------------------------------MQFEIDR-----------

1567 EAARR-------------------------------------------------------

1595 ------AENRAQR-----------------------------------------------

1607 -----------------------------------------NMQFEID------------

1615 ---------------------RIEMERR--------------------------------

1643 EAAR--------------------------------------------------------

1647 ------------------QQLRIEM-----------------------------------

1689 ----------------------------------------------IDRLTAA-------

O_lupi MKAEIARLKKKYQAEIAELEMTVDNLNRANIEAQKTIKKQSEQLKVLQASLEDTQRQLQQ

97 -----------YQAEIAE------------------------------------------

155 ----------------AELEMTV-------------------------------------

167 ---------------------------------------------VLQASLE--------

169 ----------------------------------KTIKKQS-------------------

187 ----------------------------------------------------------QQ

211 -------------------------LNRANIE----------------------------

213 ---EIARLKK--------------------------------------------------

233 ------------------------------------------QLKVLQA-----------

263 ------------------------------IEAQKTI-----------------------

297 ---------------------------RANIEAQ--------------------------

309 ----------------------VDNLNRA-------------------------------

323 ---------------------------------------------------------LQQ

347 ------RLKKKYQ-----------------------------------------------

361 ---------------------------------------QSEQLKV--------------

375 ---------------------TVDNLNR--------------------------------

417 -------LKKKYQA----------------------------------------------

423 ----IARLKKK-------------------------------------------------

461 MK----------------------------------------------------------

465 ------------------------------------------------------QRQLQQ

475 -----------------------------NIEAQKT------------------------

481 -----------------------------------------------QASLEDT------

491 MKAEI-------------------------------------------------------

495 MKA---------------------------------------------------------

499 ----------------------------ANIEAQK-------------------------

539 -------------------------------------------------SLEDTQR----

563 --------------------------------------------KVLQASL---------

591 ------------QAEIAEL-----------------------------------------

605 ----------------------------------------------LQASLED-------

657 -KAEIARL----------------------------------------------------

671 --------KKKYQAE---------------------------------------------

695 ------------------------------------------------ASLEDTQ-----

701 --------------------------------------------------LEDTQRQ---

705 ---------------------------------QKTIKKQ--------------------

763 --------------------------------------KQSEQLK---------------

773 ---------KKYQAEI--------------------------------------------

791 ----------------------------------------SEQLKVL-------------

821 ----------KYQAEIA-------------------------------------------

843 MKAE--------------------------------------------------------

935 M-----------------------------------------------------------

939 -------------AEIAELE----------------------------------------

1003 ---------------IAELEMT--------------------------------------

1121 -----------------ELEMTVD------------------------------------

1139 --------------------------------AQKTIKK---------------------

1161 ----------------------------------------------------DTQRQLQ-

1199 MKAEIAR-----------------------------------------------------

1227 MKAEIA------------------------------------------------------

1297 -----------------------------------------------------TQRQLQQ

1305 -------------------------------------------LKVLQAS----------

1319 -----------------------DNLNRAN------------------------------

1327 -----------------------------------TIKKQSE------------------

1345 -------------------------------EAQKTIK----------------------

1349 ------------------------------------IKKQSEQ-----------------

1375 --------------------MTVDNLN---------------------------------

1399 --AEIARLK---------------------------------------------------

1423 -----ARLKKKY------------------------------------------------

1445 -----------------------------------------EQLKVLQ------------

1525 ---------------------------------------------------EDTQRQL--

1547 -----------------------------------------------------------Q

1551 ------------------------NLNRANI-----------------------------

1577 -------------------EMTVDNL----------------------------------

1587 -------------------------------------KKQSEQL----------------

1637 --------------EIAELEM---------------------------------------

1703 --------------------------------------------------------QLQQ

1725 -------------------------------------------------------RQLQQ

1741 ------------------LEMTVDN-----------------------------------

O_lupi TLDQYALAQRKVSALSAELEECKVALDNAIRARKQAEIDLEEANGRIADLVSVNNNLTAI

19 ------LAQRKVS-----------------------------------------------

35 -------------------------------------IDLEEAN----------------

65 -----------------------VALDNAI------------------------------

77 -------------------------------------------NGRIADL----------

89 ----------------------------------------------------VNNNLTA-

93 ------------------------------------------ANGRIAD-----------

135 -----------------------------------------------------NNNLTAI

147 --------------------------DNAIRAR---------------------------

159 ----YALAQRK-------------------------------------------------

187 TLDQY-------------------------------------------------------

193 -------------------------------------------------------NLTAI

203 --------------LSAELEE---------------------------------------

267 ------------------------------------EIDLEEA-----------------

279 ---------------------------------------------------SVNNNLT--

323 TLDQ--------------------------------------------------------

343 ----------------------KVALDNA-------------------------------

381 ---------------------------------------LEEANGR--------------

389 -------------------EECKVAL----------------------------------

407 ----------------------------------------EEANGRI-------------

421 -----------------------------IRARKQA------------------------

465 T-----------------------------------------------------------

485 -------------------------LDNAIRA----------------------------

529 --DQYALAQ---------------------------------------------------

551 ---------------------------------------------RIADLVS--------

553 -------------ALSAELE----------------------------------------

557 --------------------------------------------------------LTAI

561 ------------SALSAEL-----------------------------------------

569 ----------------------------AIRARKQ-------------------------

585 ----------------------------------------------------------AI

627 ---QYALAQR--------------------------------------------------

635 ---------------------------NAIRARK--------------------------

693 -----------------------------------------------------------I

713 ----------------------------------------------IADLVSV-------

723 -------AQRKVSA----------------------------------------------

727 ---------------------------------------------------------TAI

855 -----ALAQRKV------------------------------------------------

923 ---------RKVSALS--------------------------------------------

963 ------------------------------RARKQAE-----------------------

993 ------------------------------------------------------NNLTAI

999 ----------------------------------QAEIDLE-------------------

1015 ------------------LEECKVA-----------------------------------

1035 -LDQYALA----------------------------------------------------

1043 -----------------ELEECKV------------------------------------

1077 --------------------------------------DLEEANG---------------

1083 ----------KVSALSA-------------------------------------------

1109 --------------------------------------------------VSVNNNL---

1157 --------------------------------------------GRIADLV---------

1159 -----------------------------------------EANGRIA------------

1291 ----------------AELEECK-------------------------------------

1347 --------QRKVSAL---------------------------------------------

1405 TLDQYAL-----------------------------------------------------

1443 -----------------------------------AEIDLEE------------------

1463 ---------------------------------KQAEIDL--------------------

1469 --------------------ECKVALD---------------------------------

1495 -----------------------------------------------ADLVSVN------

1505 -------------------------------ARKQAEI----------------------

1547 TLDQYA------------------------------------------------------

1619 ------------------------------------------------DLVSVNN-----

1653 --------------------------------RKQAEID---------------------

1661 ---------------------CKVALDN--------------------------------

1703 TLD---------------------------------------------------------

1725 TL----------------------------------------------------------

1733 ---------------SAELEEC--------------------------------------

1735 -----------VSALSAE------------------------------------------

1745 -------------------------------------------------LVSVNNN----

O_lupi KNKLETELSTAQADL**DEATKELHAADERANR**ALADAARAVEQ**LHEEQEHSMK**IDALRKSL

29 -----------------------------------AARAVEQ------------------

69 -------------------------------------------HEEQEHS----------

91 -------------------------------------RAVEQLH----------------

101 ----------------------------------------------QEHSMKI-------

193 KN----------------------------------------------------------

209 ----------------------------ANRALAD-------------------------

221 ---------------------LHAADER--------------------------------

229 ------------------------------------------LHEEQEH-----------

277 --------------------ELHAADE---------------------------------

289 --------------------------------------------EEQEHSM---------

301 ----------AQADLDE-------------------------------------------

331 ------ELSTAQA-----------------------------------------------

345 ---------------------------------------------------KIDALRK--

387 -----------------------AADERAN------------------------------

409 ---------------------------------------------------------KSL

429 --------------------------------------AVEQLHE---------------

437 -----------------------------------------QLHEEQE------------

453 ----------------------HAADERA-------------------------------

497 --------STAQADL---------------------------------------------

07 -------------------------------------------------SMKIDAL----

509 ------------------TKELHAA-----------------------------------

557 KNK---------------------------------------------------------

573 ------------------------------------------------------ALRKSL

585 KNKLE-------------------------------------------------------

593 -----TELSTAQ------------------------------------------------

597 -------------------------------ALADAAR----------------------

625 ------------------------------------------------HSMKIDA-----

643 ---------------------------RANRALA--------------------------

649 ---------------------------------------VEQLHEE--------------

669 -------------------------DERANRA----------------------------

673 --------------------------ERANRAL---------------------------

693 KNKLET------------------------------------------------------

727 KNKL--------------------------------------------------------

729 --------------LDEATKE---------------------------------------

743 ------------ADLDEAT-----------------------------------------

747 ----------------------------------------EQLHEEQ-------------

783 -----------------------------------------------------------L

811 ---------------------------------------------EQEHSMK--------

851 ----------------EATKELH-------------------------------------

909 ------------------------ADERANR-----------------------------

929 -------------------------------------------------------LRKSL

931 -------------------KELHAAD----------------------------------

967 ----------------------------------DAARAVE-------------------

993 K-----------------------------------------------------------

1013 KNKLETE-----------------------------------------------------

1063 --------------------------------LADAARA---------------------

1093 ------------------------------RALADAA-----------------------

1137 -----------------------------------------------------DALRKSL

1143 ----ETELSTA-------------------------------------------------

1147 -NKLETEL----------------------------------------------------

1217 -----------------------------NRALADA------------------------

1241 ----------------------------------------------------------SL

1247 ----------------------------------------------------IDALRKS-

1325 ---------------------------------ADAARAV--------------------

1365 ---------TAQADLD--------------------------------------------

1381 -------------DLDEATK----------------------------------------

1419 --------------------------------------------------------RKSL

1435 -------LSTAQAD----------------------------------------------

1477 ---LETELST--------------------------------------------------

1479 ------------------------------------ARAVEQL-----------------

1545 --KLETELS---------------------------------------------------

1589 ---------------DEATKEL--------------------------------------

1635 -----------------------------------------------EHSMKID------

1731 -----------QADLDEA------------------------------------------

1743 --------------------------------------------------MKIDALR---

O_lupi EEQVKQLQVQIQEAEAAALLGGKRVIAKLETRIRDLETALDEETRRHKETQGALRKKDRR

7 -------------------------------------TALDEET----------------

13 ---------------------GKRVIAK--------------------------------

17 ----------------------------------------------------ALRKKDR-

33 -----------------ALLGGKR------------------------------------

39 ----------------AALLGGK-------------------------------------

139 ------------------------------------ETALDEE-----------------

173 ----------IQEAEAA-------------------------------------------

253 -------------AEAAALL----------------------------------------

269 ----------------------KRVIAKL-------------------------------

355 --------------------------AKLETRI---------------------------

399 ---------------------------------------------------------DRR

409 EEQV--------------------------------------------------------

435 -----------------------------ETRIRDL------------------------

459 ----------------------------------------------HKETQGA-------

463 --------------------------------------ALDEETR---------------

483 -------------------------------------------------------KKDRR

559 ------LQVQIQE-----------------------------------------------

567 -----------------------------------LETALDE------------------

573 E-----------------------------------------------------------

589 ------------------------------------------------ETQGALR-----

599 --------------------GGKRVIA---------------------------------

617 -----------------------------------------------------------R

623 ------------------------------------------ETRRHKE-----------

659 --------------------------------IRDLETA---------------------

661 -----------------------------------------------------LRKKDRR

667 --------VQIQEAE---------------------------------------------

765 -----------------------RVIAKLE------------------------------

783 EEQVKQ------------------------------------------------------

815 ---VKQLQVQ--------------------------------------------------

831 EEQVKQL-----------------------------------------------------

839 --------------EAAALLG---------------------------------------

863 -------------------------------------------TRRHKET----------

887 ----KQLQVQI-------------------------------------------------

901 ------------------------------TRIRDLE-----------------------

905 ------------------LLGGKRV-----------------------------------

929 EE----------------------------------------------------------

973 ---------------------------------------------------GALRKKD--

981 -------------------------------------------------TQGALRK----

1037 ------------EAEAAAL-----------------------------------------

1059 --------------------------------------------------------KDRR

1089 ---------------------------------RDLETAL--------------------

1091 ------------------------------------------------------RKKDRR

1115 ---------QIQEAEA--------------------------------------------

1123 -------QVQIQEA----------------------------------------------

1177 --QVKQLQV---------------------------------------------------

1183 ----------------------------------------DEETRRH-------------

1191 ----------------------------------DLETALD-------------------

1213 -----------------------------------------EETRRHK------------

1225 ----------------------------------------------------------RR

1229 -----------------------------------------------KETQGAL------

1241 EEQVK-------------------------------------------------------

1259 --------------------------------------------------QGALRKK---

1281 ---------------------------KLETRIR--------------------------

1295 --------------------------------------------RRHKETQ---------

1419 EEQ---------------------------------------------------------

1431 ---------------AAALLGG--------------------------------------

1457 -EQVKQLQ----------------------------------------------------

1497 -------------------------IAKLETR----------------------------

1513 ------------------------VIAKLET-----------------------------

1521 -----QLQVQIQ------------------------------------------------

1549 -------------------------------RIRDLET----------------------

1563 -----------QEAEAAA------------------------------------------

1611 -------------------LGGKRVI----------------------------------

1657 ----------------------------LETRIRD-------------------------

1681 ---------------------------------------------RHKETQG--------

1697 ---------------------------------------LDEETRR--------------

O_lupi IK**EVQMQVDEE**HKMFVMAQDTADRLLEKLNIQKR**QLGE**AESLTMANLQRVRRYQRELEDA

15 -------------------------LEKLNIQ----------------------------

31 -------VDEEHKM----------------------------------------------

47 ---------------------------KLNIQKR--------------------------

111 ------------------------------------------------------RELEDA

115 ---------------------------------RQLGEAE--------------------

157 ----QMQVDEE-------------------------------------------------

219 ---------------------------------------ESLTMAN--------------

257 ---VQMQVDE--------------------------------------------------

317 -------------------------------------------MANLQRV----------

359 --------------------------------------------ANLQRVR---------

367 --------------------------------------AESLTMA---------------

373 ---------------------ADRLLEK--------------------------------

399 IKEV--------------------------------------------------------

451 ----------------------------------------------------------DA

471 --------------------TADRLLE---------------------------------

483 IK----------------------------------------------------------

617 IKEVQM------------------------------------------------------

621 -------------------------------------EAESLTM----------------

711 ----------------------DRLLEKL-------------------------------

789 ----------------MAQDTAD-------------------------------------

847 -----------------------------------------------QRVRRYQ------

879 -----------------------------------------------------------A

883 -----------------------------------LGEAESL------------------

895 --------------------------------------------------------LEDA

937 -----MQVDEEH------------------------------------------------

941 -----------------------------------------LTMANLQ------------

943 ---------------------------------------------------RYQRELE--

959 -------------------------------------------------VRRYQRE----

961 ------------------------------------------TMANLQR-----------

979 -------------------DTADRLL----------------------------------

1017 ----------------------------LNIQKRQ-------------------------

1029 --------DEEHKMF---------------------------------------------

1039 ------------------------------------------------RVRRYQR-----

1059 IKE---------------------------------------------------------

1061 ------------------QDTADRL-----------------------------------

1087 ---------------------------------------------NLQRVRR--------

1091 I-----------------------------------------------------------

1107 --------------------------EKLNIQK---------------------------

1209 ---------------------------------------------------------EDA

1211 ----------EHKMFVM-------------------------------------------

1225 IKEVQ-------------------------------------------------------

1233 -KEVQMQV----------------------------------------------------

1249 -------------------------------QKRQLGE----------------------

1271 -----------------------------NIQKRQL------------------------

1293 --------------FVMAQDT---------------------------------------

1323 ------QVDEEHK-----------------------------------------------

1333 IKEVQMQ-----------------------------------------------------

1337 ----------------------------------------------------YQRELED-

1397 --------------------------------------------------RRYQREL---

1413 ----------------------------------------------LQRVRRY-------

1421 -----------------AQDTADR------------------------------------

1465 ---------EEHKMFV--------------------------------------------

1471 -----------HKMFVMA------------------------------------------

1487 --------------------------------KRQLGEA---------------------

1493 -------------MFVMAQD----------------------------------------

1523 -----------------------RLLEKLN------------------------------

1555 ------------------------LLEKLNI-----------------------------

1569 ----------------------------------QLGEAES-------------------

1599 ------------KMFVMAQ-----------------------------------------

1605 ------------------------------------GEAESLT-----------------

1609 ---------------VMAQDTA--------------------------------------

1639 ------------------------------IQKRQLG-----------------------

1673 ----------------------------------------SLTMANL-------------

1683 -----------------------------------------------------QRELEDA

O_lupi EGRPDQAESSLHLIRAKHRSSVVSGKNASASKIYVLEDEQ

45 ----DQAESSL-------------------------------------

75 ------------------------------SKIYVLE-----------

111 E-----------------------------------------------

149 ------------LIRAKHR-----------------------------

217 ----------LHLIRAK-------------------------------

283 ------------------RSSVVSG-----------------------

451 EGRPD-------------------------------------------

479 ---------------AKHRSSV--------------------------

681 --------------------------NASASKI---------------

707 -------------------SSVVSGK----------------------

709 -GRPDQAE----------------------------------------

767 -------------IRAKHRS----------------------------

823 -------ESSLHLI----------------------------------

879 EGRPDQ------------------------------------------

895 EGR---------------------------------------------

947 ----------------------VSGKNAS-------------------

969 ---------SLHLIRA--------------------------------

977 EGRPDQA-----------------------------------------

985 -------------------------------KIYVLED----------

1025 -----------------------------ASKIYVL------------

1103 ------------------------GKNASAS-----------------

1187 --------SSLHLIR---------------------------------

1209 EGRP--------------------------------------------

1219 -----------HLIRAKH------------------------------

1287 -----QAESSLH------------------------------------

1299 -----------------------SGKNASA------------------

1313 ----------------KHRSSVV-------------------------

1315 --RPDQAES---------------------------------------

1321 ---PDQAESS--------------------------------------

1403 ---------------------------ASASKIY--------------

1407 --------------RAKHRSS---------------------------

1537 -------------------------KNASASK----------------

1561 ----------------------------SASKIYV-------------

1621 ---------------------VVSGKNA--------------------

1625 -----------------HRSSVVS------------------------

1695 --------------------SVVSGKN---------------------

1709 ------AESSLHL-----------------------------------

1721 --------------------------------IYVLEDE---------
